# Investigation and genome-wide association study for Fusarium crown rot resistance in Chinese common wheat

**DOI:** 10.1186/s12870-019-1758-2

**Published:** 2019-04-23

**Authors:** Xia Yang, Yubo Pan, Pawan K. Singh, Xinyao He, Yan Ren, Lei Zhao, Ning Zhang, Shunhe Cheng, Feng Chen

**Affiliations:** 1 Agronomy College/National Key Laboratory of Wheat and Maize Crop Science/Collaborative Innovation Center of Henan Grain Crops, Henan Agricultural University, 15 Longzihu College District, Zhengzhou, 450046 China; 2 International Maize and Wheat Improvement Center (CIMMYT), Apdo. Postal 6-641, 06600 Mexico DF, Mexico; 3 Lixiahe Institute of Agricultural and Sciences, Yangzhou, 225007 Jiangsu China

**Keywords:** Bread wheat, Fusarium crown rot, Disease index, GWAS, QTL

## Abstract

**Background:**

Fusarium crown rot (FCR) is a severe and chronic disease in common wheat and is able to cause serious yield loss and health problems to human and livestock.

**Results:**

Here, 234 Chinese wheat cultivars were evaluated in four greenhouse experiments for FCR resistance and genome-wide association studies (GWAS) were performed using the wheat 660 K genotyping assay. The results indicated that most cultivars evaluated showed FCR disease index (DI) of 40–60, while some cultivars showed stably good FCR resistance (DI < 30). GWAS identified 286 SNPs to be significantly associated with FCR resistance, of which 266, 6 and 8 were distributed on chromosomes 6A, 6B and 6D, respectively. The significant SNPs on 6A were located in a 7.0-Mb region containing 51 annotated genes. On the other hand, QTL mapping using a bi-parental population derived from UC1110 and PI610750 detected three QTLs on chromosomes 6A (explaining 7.77–10.17% of phenotypic variation), 2D (7.15–9.29%) and 2A (5.24–6.92%). The 6A QTL in the UC1110/PI610750 population falls into the same chromosomal region as those detected from GWAS, demonstrating its importance in Chinese materials for FCR resistance.

**Conclusion:**

This study could provide useful information for utilization of FCR-resistant wheat germplasm and further understanding of molecular and genetics basis of FCR resistance in common wheat.

**Electronic supplementary material:**

The online version of this article (10.1186/s12870-019-1758-2) contains supplementary material, which is available to authorized users.

## Background

Fusarium crown rot (FCR), also known as foot rot or root rot, is one of the most seriously insidious disease of wheat and barley [1, 2]. This disease is mainly caused by the fungal pathogen *F. Pseudograminearum*, which often co-exists with other FCR-causing *Fusarium* species, such as *F. graminearum*, *F. culmorum* and *F. avenaceum* [3]. Infected seedlings are usually characterized by browning in coleoptile, leaf sheath and stem base, which can become evident after planting and throughout plant development [4].

FCR is widespread in many parts of the arid and semi-arid regions of the world and is of economic concern in Australia, Canada, the Pacific Northwest of the USA, North Africa, South Africa, China and the Middle East [4]. In Australia, FCR caused an estimated annual yield loss of $97 million Australian dollars in wheat and barley [5, 6], while in the Pacific Northwest of the USA, it reduced yields of winter wheat by up to 35% and on barely an average of 13% in commercial fields [7]. In addition, wheat grains infected by *Fusarium* species were usually associated with the accumulation of mycotoxins like deoxynivalenol (DON) [8] and nivalenol (NIV) [9], which are harmful to human and livestock [10].

In China, wheat is one of the main food crops accounting for a considerable proportion of planting area and crop production. The Yellow and Huai wheat region, with 60–70% of both total harvested area and production, is the largest and most important wheat production zone in China [11]. In recent years, wheat FCR has rapidly increased in this region, including main production regions of Henan and Shandong, south-central Hebei, northern Anhui and Jiangsu, southern Shanxi and east-central Shaanxi [12]. According to Li et al. [13], more than 10% planting areas in Henan province were affected with FCR and more than 30% of yield loss was reported in Xuchang, Jiaozuo and other epidemic places. FCR has occurred seriously in Xinxiang with an incidence area of 3334 ha and yield loss of 10–20%, even exceeding 50% in some sites [14]. Meanwhile, previous studies showed that wheat germplasm with high degree of FCR resistance are rare [4], and almost all current popular varieties in the Yellow and Huai wheat region are susceptible or highly susceptible to FCR [15]. Therefore, developing FCR-resistant cultivars has been vital to prevent the yield damage in cereals [16, 17].

Significant progress in identifying FCR-resistance QTL has been made in wheat and barley. There are three large effect QTLs on the long arms of chromosomes 1H, 3H and 4H in barley [18, 19]. According to the research from CIMMYT and other research institutes, FCR QTLs conferring partial resistance have been identified in different wheat varieties worldwide [20]. These QTLs are distributed on 13 of the 21 wheat chromosomes [2]. One of the most important FCR-resistant QTL is located on 3BL in a RIL population of ‘CSCR6/Lang’ and explained the phenotypic variation up to 49% [21], and limited data further shows that it confers field resistance to FCR and reduces whitehead incidence. The QTL on 4B identified from a cross of ‘Kukri/Janz’ was near the dwarfing gene *Rht1* [22] and this QTL showed limited effect of FCR resistance. Additional studies have identified significant QTLs on various chromosomes, e.g. 2B, 3B, 4B, 4D and 7A in RIL populations ‘Sunco/Macon’ and ‘Sunco/Otis’ [23], and QTLs on 2D and 5D in a RIL population ‘Wylie/Sumai 3’ [24]. Genomic analysis of FCR resistance in bread wheat revealed 6, 25 and 11 FCR-resistant QTLs in the A, B and D sub-genome, respectively [2].

As an effective method to study the associations between nucleotide polymorphisms and phenotypic variation: GWAS which has been widely used for analyzing the inheritance of agronomic traits and disease resistance [25, 26]. The newly developed wheat 660 K SNP genotyping assay is generally more abundant and effective than the 90 K SNP [27]. The advances in high-throughput sequencing technologies have enabled rapid and accurate sequencing of a large number of genomes and facilitated direct searches for the causal variation underlying phenotypic diversity [28, 29]. To date, GWAS has been conducted in many plant species, including Arabidopsis, maize, rice, barley, sorghum and wheat [30–32]. In wheat, GWAS has been used to study agronomic traits, quality traits and disease resistance [33]. In a previous study, Sun et al. [34] used GWAS to investigate the distribution of superior alleles of 13 agronomic traits in bread wheat from the Yellow and Huai wheat region of China. Marco et al. [33] used GWAS to map strip rust resistance QTL in a worldwide collection of hexaploid spring wheat. In the research by Gurung et al. [35], novel QTL associated with resistance to multiple leaf spot diseases were revealed by GWAS analysis. GWAS for wheat FCR has rarely been reported, and the molecular mechanisms for FCR remain poorly understood.

The Yellow and Huai wheat region is the largest and most important wheat production zone of China [11]. Varieties in this region play a major role in national wheat production. FCR is becoming more prevalent in this region due to a lack of varietal resistance. In this study, we evaluated FCR resistance in bread wheat accessions from the Yellow and Huai wheat region, and then performed GWAS and bi-parental QTL analysis. Our aims were: 1) to identify FCR-resistant wheat germplasms that could be used as resistance donors in breeding, 2) to uncover novel FCR-resistant loci that could be used in marker-assisted selection, and 3) to provide useful information for understanding of molecular and genetic basis of FCR resistance in common wheat.

## Results

### Resistance investigation of Fusarium crown rot

**Fig. 1:**
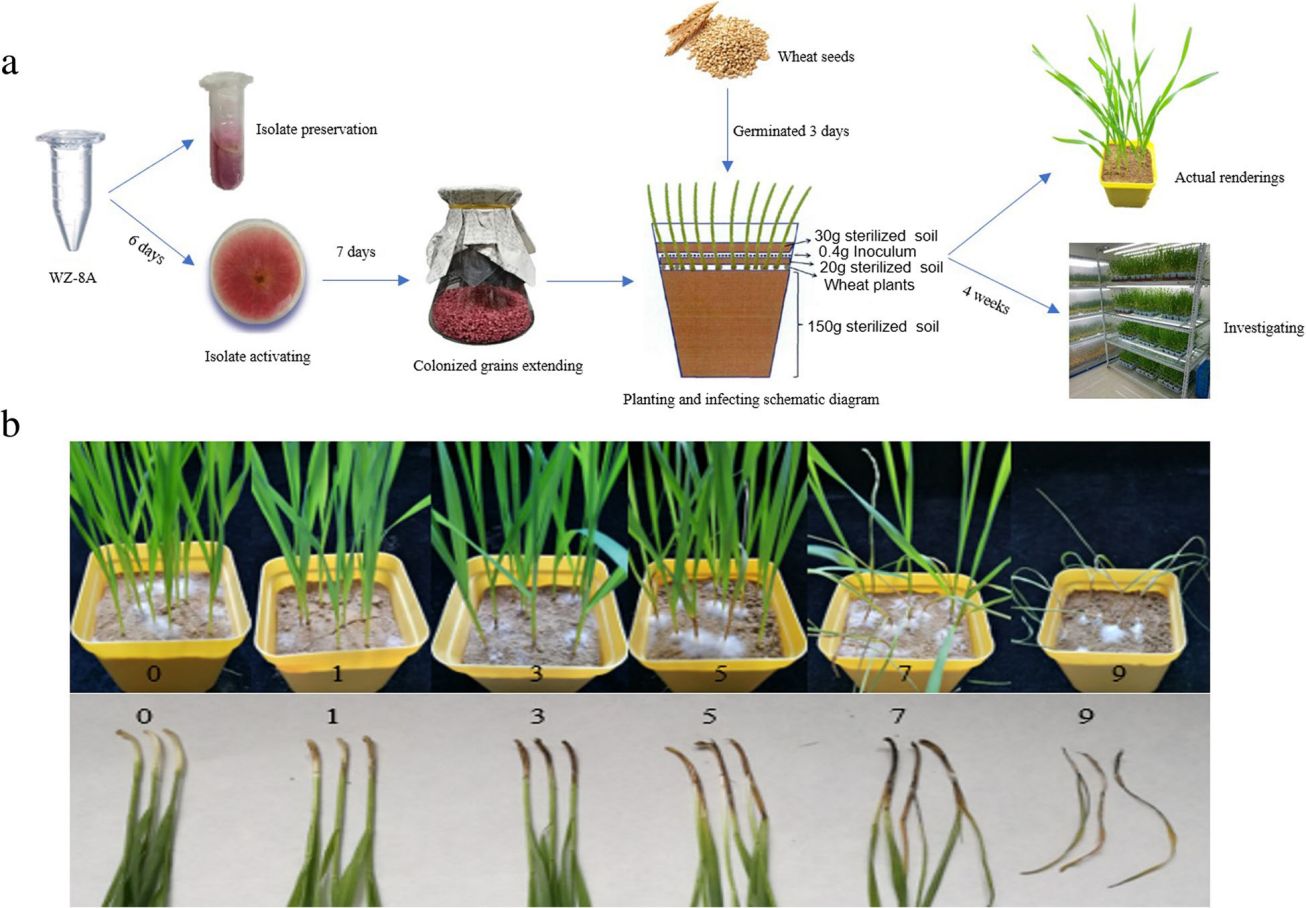
Schematic diagram for FCR inoculation and screening. (**a**) Isolate preservation, experimental design and inoculation; (**b**) typical FCR symptoms on seedlings in greenhouse experiments, scaling from 0 to 9

**Fig. 2:**
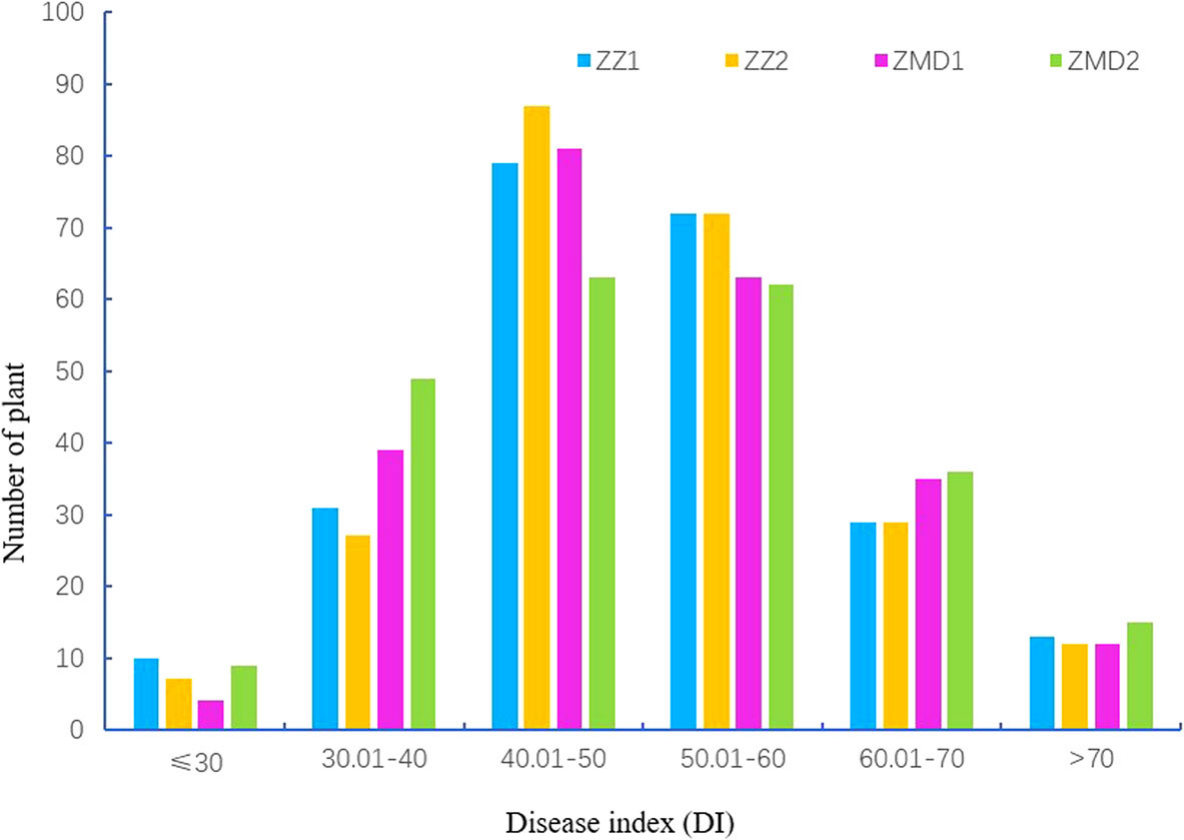
Number of cultivars at different levels of resistance to FCR in the four environments. ZZ1, ZZ2, ZMD1, ZMD2 represent 2015–2016 Zhengzhou, 2016–2017 Zhengzhou, 2015–2016 Zhumadian and 2016–2017 Zhumadian, respectively

The schematic diagram of FCR inoculation and resistance investigation was shown in Fig. 1. The assessment of FCR resistance in Chinese common wheat showed a very broad range of DI from 20.6 to 84.4 with a normal distribution in the four environments (Additional file 1: Table S1, Fig. 2). Correlation coefficients among the four environments ranged from 0.71 to 0.95 (Additional file 2: Table S2). ANOVA showed that environment had no significant effect on the seedling FCR resistance in the panel (Additional file 3: Table S3). Further analysis indicated that only 7 accessions (Yanke 316, Xunmai 118, Kaimai 26, Jiyanmai 7, Zhonglemai 9, Shenzhou 209 and Jinmai 1) showed an averaged DI of less than 30, exhibiting their potentiality in wheat FCR resistance breeding. There were 31, 87, 70, 27 and 11 accessions showing averaged DI ranges of 30.01–40, 40.01–50, 50.01–60, 60.01–70 and 70.01–80, respectively, and only one cultivar (Zhengmai 082) showed an averaged DI of more than 80. Results indicated that majority of the varieties fell into the range of 40.01–60 (67.09%), indicating an urgent need of improving FCR resistance in the Yellow and Huai wheat region. Representative materials with different grades of resistance to FCR were showed in Table 1.

**Table 1:** Materials with different resistance levels to FCR (only representative materials are shown)

Disease index	Cultivars
≤ 30	Xunmai 118, Kaimai 26, Yanke 316, Xuke 732, Zhonglemai 9, Jinmai 1, Shenzhou 209, Fannong 1, Jiyanmai 7
30.01–40	Xianmai 522, Fumai 188, L 668, Xinyanmai 98, Zhonglemai 9, Xuke 732, Jiangmai 816, Zhoukang 918, Ximai 505, Yufeng 1, Gengmai 237, Zimai 627, Xinzhi 519, Fengmai 52, Jinmai 108, Xinmai 37, Zhengxin 758, Xuke 877, Huimai 216, Fengdecunmai 19, Chuangxin 10
40.01–60	Defeng 108, Fannong 3, Pumai 27, Zhumai 706, Nongda 399, Yunong 99, Xuyou 46, Neimai 6, Hengmai 18, Zhengke 6, Wanmai 99, Zhengmai 516, Xuyan 2, Bainong 1309, Luomai 166, Zhouyumai 36, Jiamai 6, Yufeng 2, Zhengda 101, Keyu 368, Jinfeng 205, Wanmai 99, Aomai 18, Bainong 1309, Yunong 169, Luomai 32, Shunmai 8, Xianhong 169, Yanmai 9719, Xuke 158, Lunxuan 163, Bainong 219, Shunmai 8
60.01–70	Luo 1807, Jimai 210, Xianyuan 988, Fanyumai 18, Qiule 2126, Xinxuan 16, Yunong 804, Jiamai 99, Jinmai 18, Kaimai 27, Yanmai 988, Taipingyuan 007, Jinwoye 1, Tianle 6, Xinong 18, Junsui1 88
> 70	Jingyumai 1, Wenmai 29, Wenyuan 0528, Songmai 518, Yumai 117, Dongfanghongmai 6, Yanfeng 712, Jinmai 109, Yunong 805, Saidemai 7, Zhengmai 082, Lunxuan 169

### Genome-wide association study

**Fig. 3:**
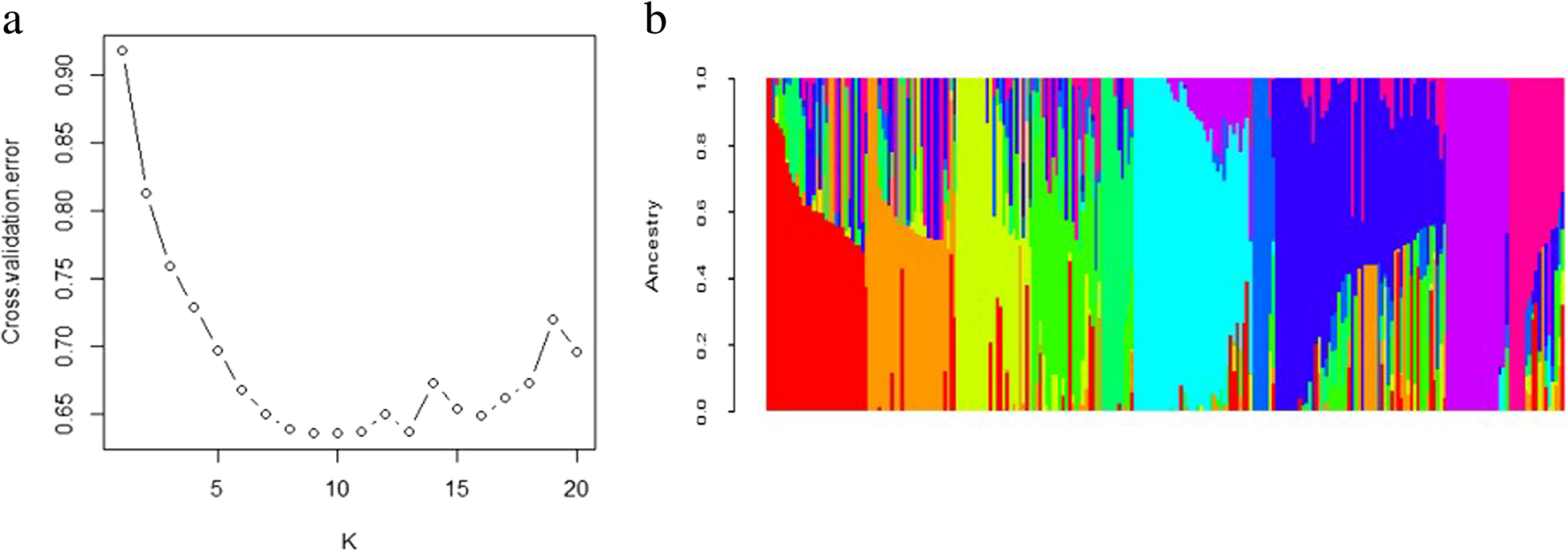
Population structure and principal component analysis (PCA) of the 234 wheat cultivars. (**a**) plot of cross. validation. error; (**b**) stacked bar plot of ancestry relationship of the 234 cultivars

**Fig. 4:**
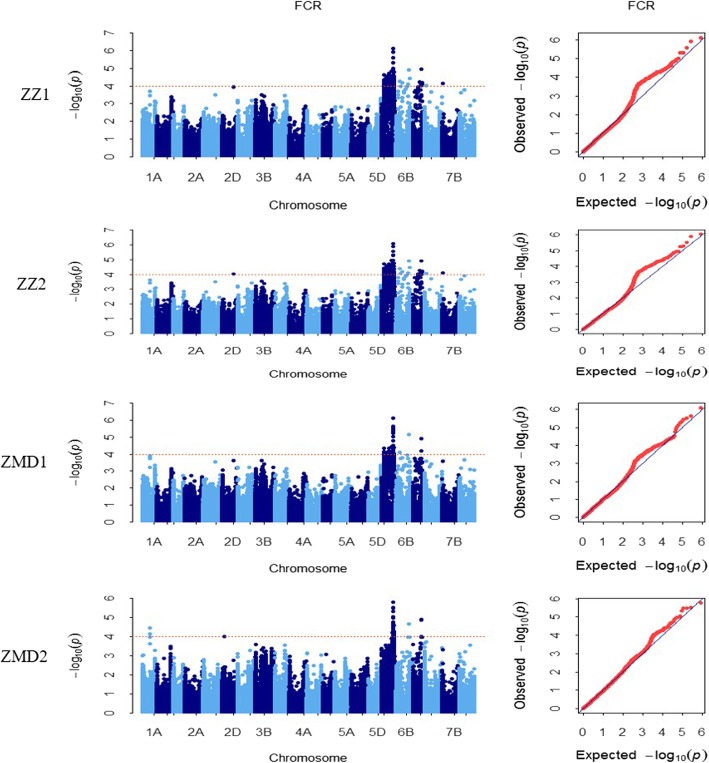
Manhattan and Q-Q plots for FCR identified by genome-wide association study (GWAS) in four environments, respectively

**Fig. 5:**
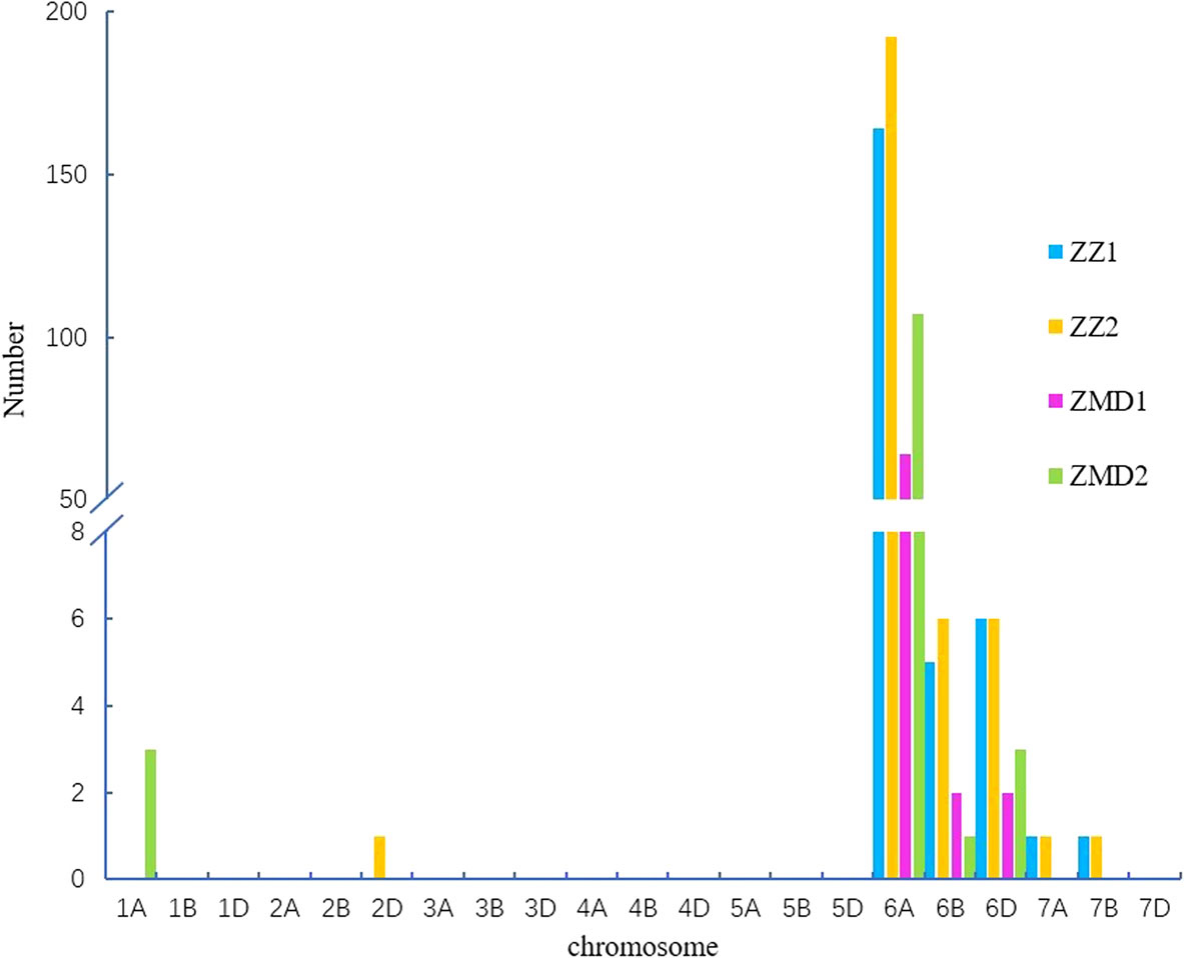
Distribution of significant SNPs revealed by GWAS on various chromosomes

**Fig. 6:**
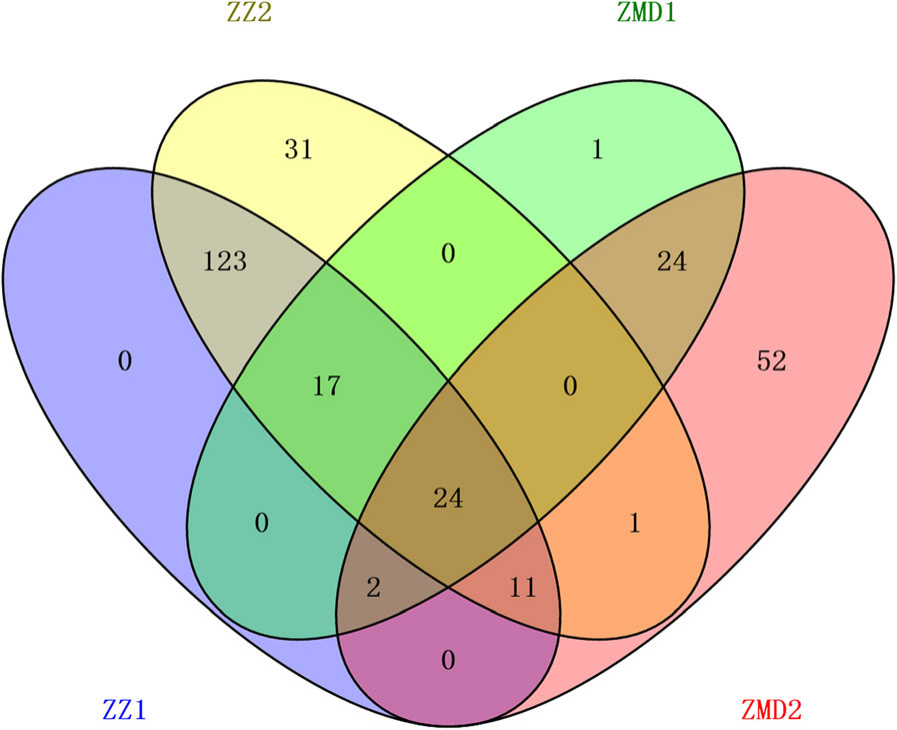
The significant SNP intersections among the four environments

**Fig. 7:**
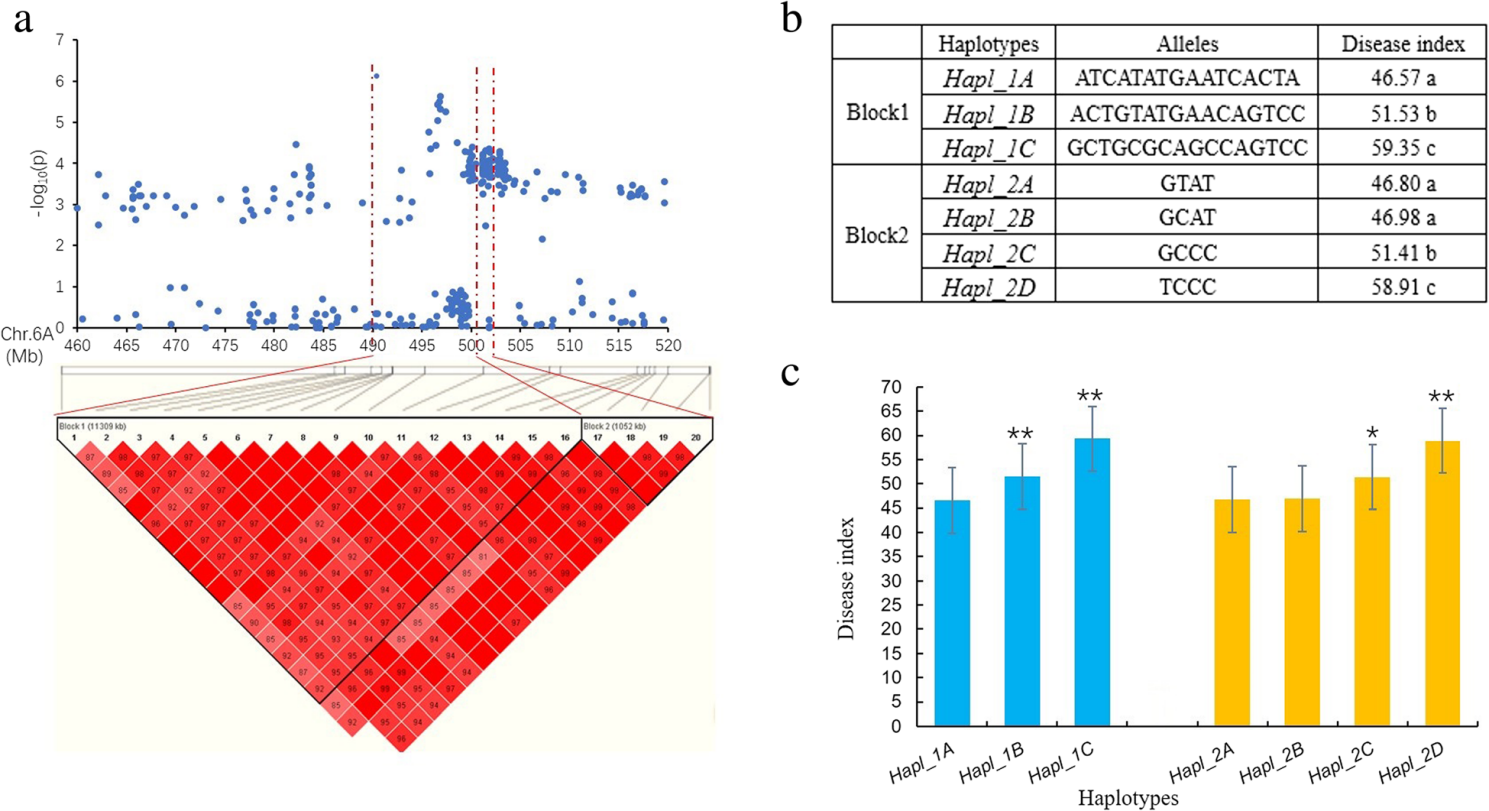
Haplotype analysis of the repeatable SNPs on chromosome 6A. (**a**) local Manhattan plot (top) and LD heatmap (bottom) surrounding the peak on chromosome 6A; (**b**) haplotypes with different alleles in the two blocks; (**c**) phenotypic effects of different haplotypes

The structure results were integrated by uploading to Structure Harvester http://taylor0.biology.ucla.edu/structureHarvester/. Figure 3 (a, b) shows that peak of the broken line graph was observed at *k* = 10, indicating ten subpopulations were observed. After filtering, 395,783 SNPs were used for GWAS analysis, and a total of 286 significant SNPs was identified from seven chromosomes, of which 266, 6 and 8 were distributed on 6A, 6B and 6D, respectively (Table 2; Figs. 4 and 5). Phenotypic variation explained by these SNPs ranged from 9.89 to 15.16% for SNPs on 6A, from 10.17 to 12.61% for SNPs on 6B and from 9.90 to 12.39% for SNPs on 6D. There were 24 SNPs being significant in all of the 4 environments (Fig. 6), and 22 of them were detected on 6A. Haplotype analysis showed that these SNPs were clustered into two blocks, where block 1 comprised 16 SNPs forming 3 haplotypes and block 2 comprised 4 SNPs forming 4 haplotypes (Fig. 7a, b). In block 1, the three haplotypes showed significantly different DI, where *Hapl_1A* showed the lowest averaged DI (46.57), followed by *Hapl_1B* (51.53) and *Hap_1C* (59.35) (Fig. 7b, c). In block 2, the four haplotypes also showed significantly different DI, where *Hapl_2A* and *Hapl_2B* showed low DI (46.8 and 46.98, respectively), followed by *Hapl_2C* (51.41) and *Hapl_2D* (58.91) (Fig. 7b, c). BLAST of the significant SNPs on 6A in the genome database of Chinese Spring showed that these SNPs ranged from 490,486,046 to 497,462,135 (≈ 7.0 Mb) containing 51 annotation genes.

**Table 2:** QTLs for FCR resistance identified in the genome-wide association study panel

Environment^a^	Chr.^b^	No. of SNPs	QTL range	MSS^b^ position	MSS *p*-value	R^2^ (%)^c^
ZZ1	6A	164	107,685,021–526,027,254	490,486,046	7.46E-07	15.16
	6B	5	175,341,814–574,019,155	534,514,143	1.25E-05	12.28
	6D	6	263,706,656–383,753,969	354,819,336	1.12E-05	12.38
	7A	1	76,851,404	76,851,404	9.62E-05	10.26
	7B	1	51,599,075	51,599,075	7.33E-05	10.52
ZZ2	2D	1	506,779,377	506,779,377	9.75E-05	10.35
	6A	192	107,385,413–526,027,254	490,486,046	8.52E-07	15.12
	6B	6	175,341,814–574,019,155	534,514,143	1.29E-05	12.35
	6D	6	212,802,484–383,753,969	354,819,336	1.23E-05	12.39
	7A	1	76,851,404	76,851,404	8.64E-05	10.47
	7B	1	51,599,075	51,599,075	7.92E-05	10.55
ZMD1	6A	64	107,691,898–525,943,319	490,486,046	7.91E-07	14.86
	6B	2	175,341,814–534,514,143	534,514,143	7.08E-06	12.61
	6D	2	354,819,336–358,976,581	354,819,336	1.22E-05	12.06
ZMD2	1A	3	297,707,126–297,712,375	297,707,230	7.44E-05	10.88
	6A	107	456,783,268–526,027,254	490,486,046	1.63E-06	14.03
	6B	1	534,514,143	534,514,143	2.21E-05	11.37
	6D	3	354,819,336–360,329,647	354,819,336	1.25E-05	11.95

^a^ZZ1, 2015-2016Zhengzhou; ZZ2, 2016-2017Zhengzhou; ZMD1, 2015-2016Zhumadian; ZMD2, 2016-2017Zhumadian

^b^Most significant SNP

^c^R square of model with SNP

**Fig. 8:**
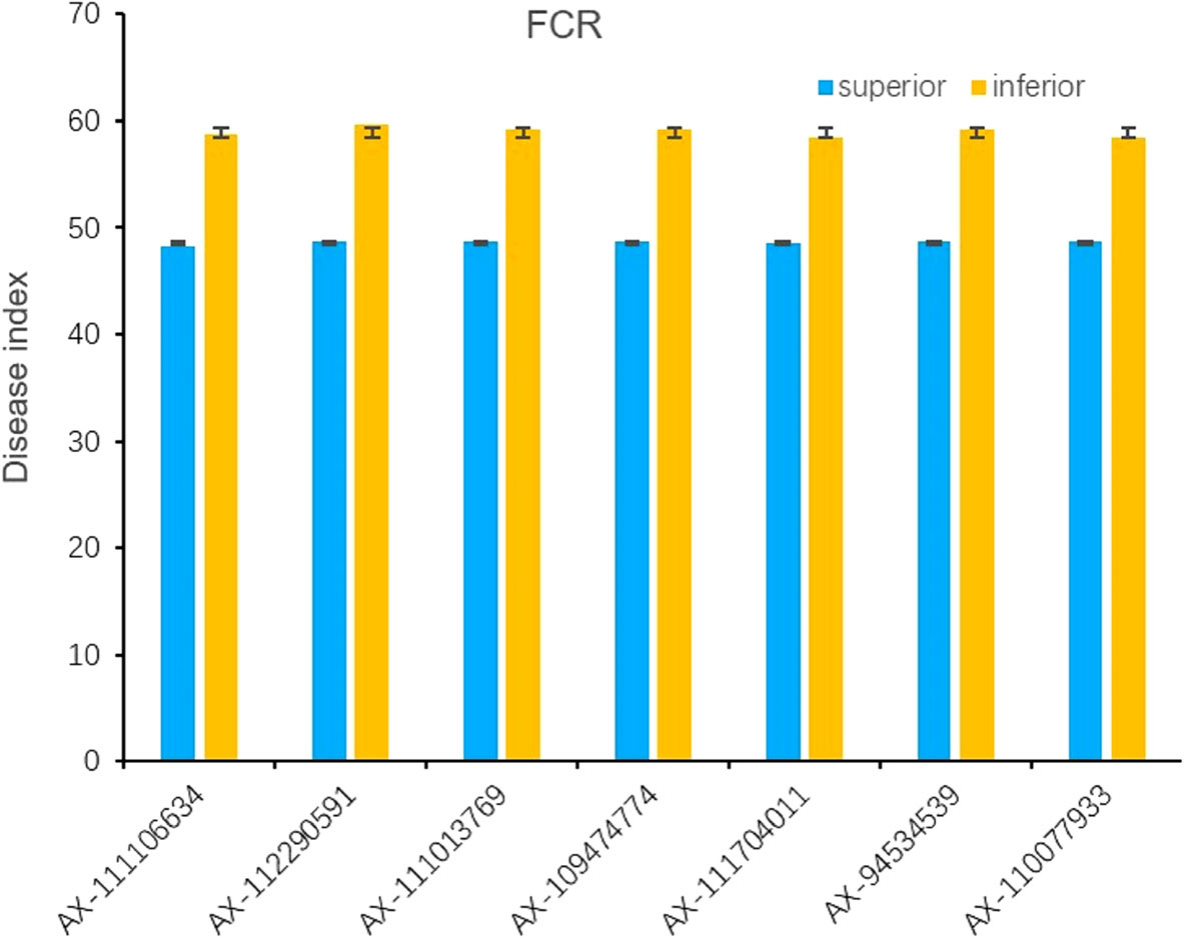
The phenotype values of cultivars with superior alleles and inferior alleles in significant loci for FCR

The repeatable SNPs which were detected in at least two environments were verified based on their polymorphism in the natural population, and the differences of phenotypic values of these SNPs reached highly significant levels in each environment (*p* < 0.01) (Table 3). Of all these SNPs, AX-111106634, AX-94534539, AX-111704011, AX-109474774, AX-112290591, AX-111013769 and AX-110077933 showed a mean phenotypic variation explained of 14.79, 13.01, 13.19, 13.28, 14.12, 13.62 and 12.31%, respectively (Additional file 4: Table S4). Cultivars with allele AA at AX-111106634 locus, allele GG at AX-112290591, allele TT at AX-111013769, allele AA at AX-94534539, allele AA at AX-111704011, allele AA at AX-109474774 and allele TT at AX-110077933 all showed relatively higher FCR resistance, respectively, and they are regarded as superior alleles (Table 3). At these seven loci, cultivars with all superior alleles showed the averaged DI of 48.58 (ranging from 48.15 to 48.70), while cultivars with all inferior alleles showed the averaged DI of 59.02 (ranging from 57.72 to 60.68) (Fig. 8). Of all varieties surveyed, six (Yanke 316, Jiyanmai 7, Jinmai 1, Zhonglemai 9, Shenzhou 209 and Fannong 1) with all these 7 superior alleles showed DI lower than 30, whereas six (Yunong 805, Zhengmai 082, Saidemai 7, Dongfanghongmai 6, Jinmai 109 and Wenmai 29) with all these 7 inferior alleles showed DI higher than 70.

**Table 3:** *P* values of *t*-test for repetitive candidate SNPs for FCR in different environments

SNP name	Chr.^a^	Allele		Number		DI		ZZ1	ZZ2	ZMD1	ZMD2
AX-111106634	6A	AA	GG	193	33	48.32b	58.73a	0.0001	0.0000	0.0000	0.0000
AX-112290591	6A	AA	GG	30	199	59.69a	48.65b	0.0000	0.0000	0.0001	0.0000
AX-111013769	6A	CC	TT	32	199	59.22a	48.65b	0.0001	0.0001	0.0000	0.0000
AX-109474774	6A	AA	GG	196	32	48.67b	58.81a	0.0001	0.0001	0.0001	-^b^
AX-111704011	6A	AA	GG	198	32	48.56b	58.43a	0.0002	0.0002	0.0001	0.0000
AX-94534539	6A	AA	CC	198	29	48.65b	59.16a	0.0002	0.0002	0.0001	0.0000
AX-110077933	6A	CC	TT	32	199	59.07a	48.57b	–	–	0.0001	0.0000

^a^The chromosome location of the SNP

^b^Indicated that data was missing

### Bi-parental QTL mapping for FCR

**Fig. 9:**
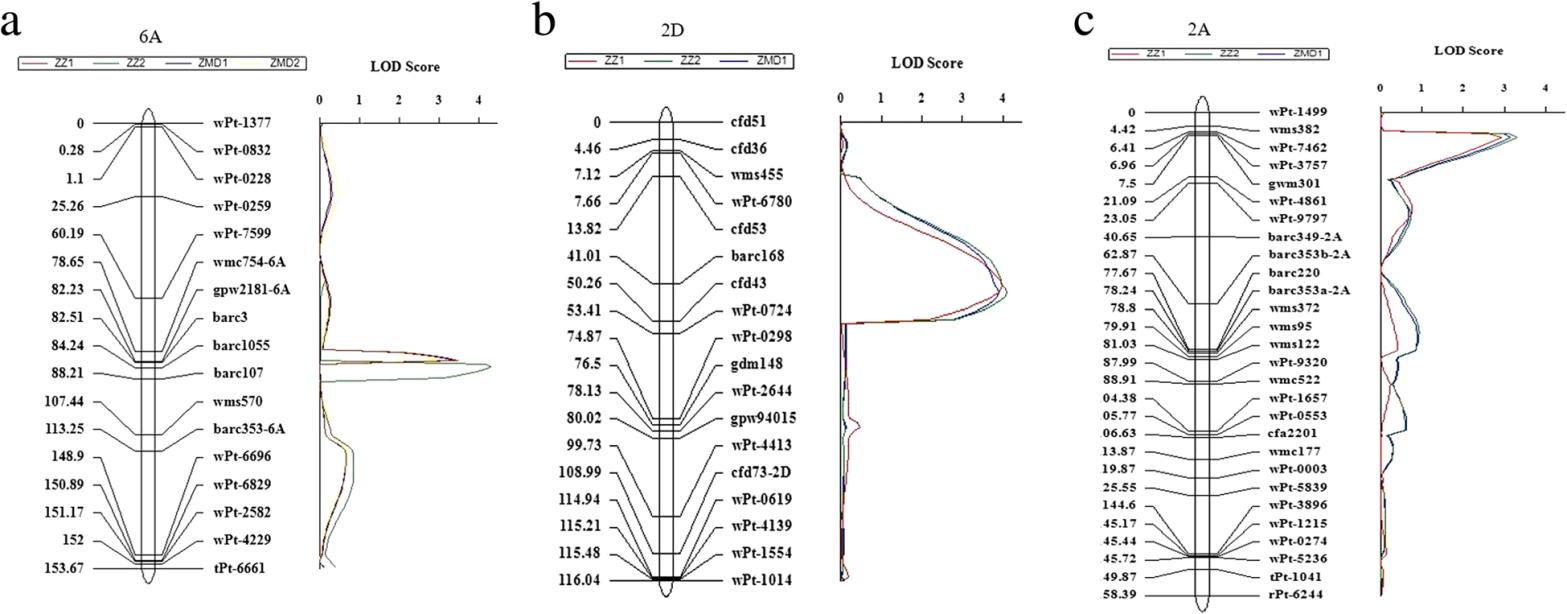
LOD contours for FCR QTL on chromosomes 6A, 2D and 2A identified in the UC1110 × PI610750 population. The significant LOD thresholds were calculated from 1000 permutations and finally set at 2.5

**Fig. 10:**
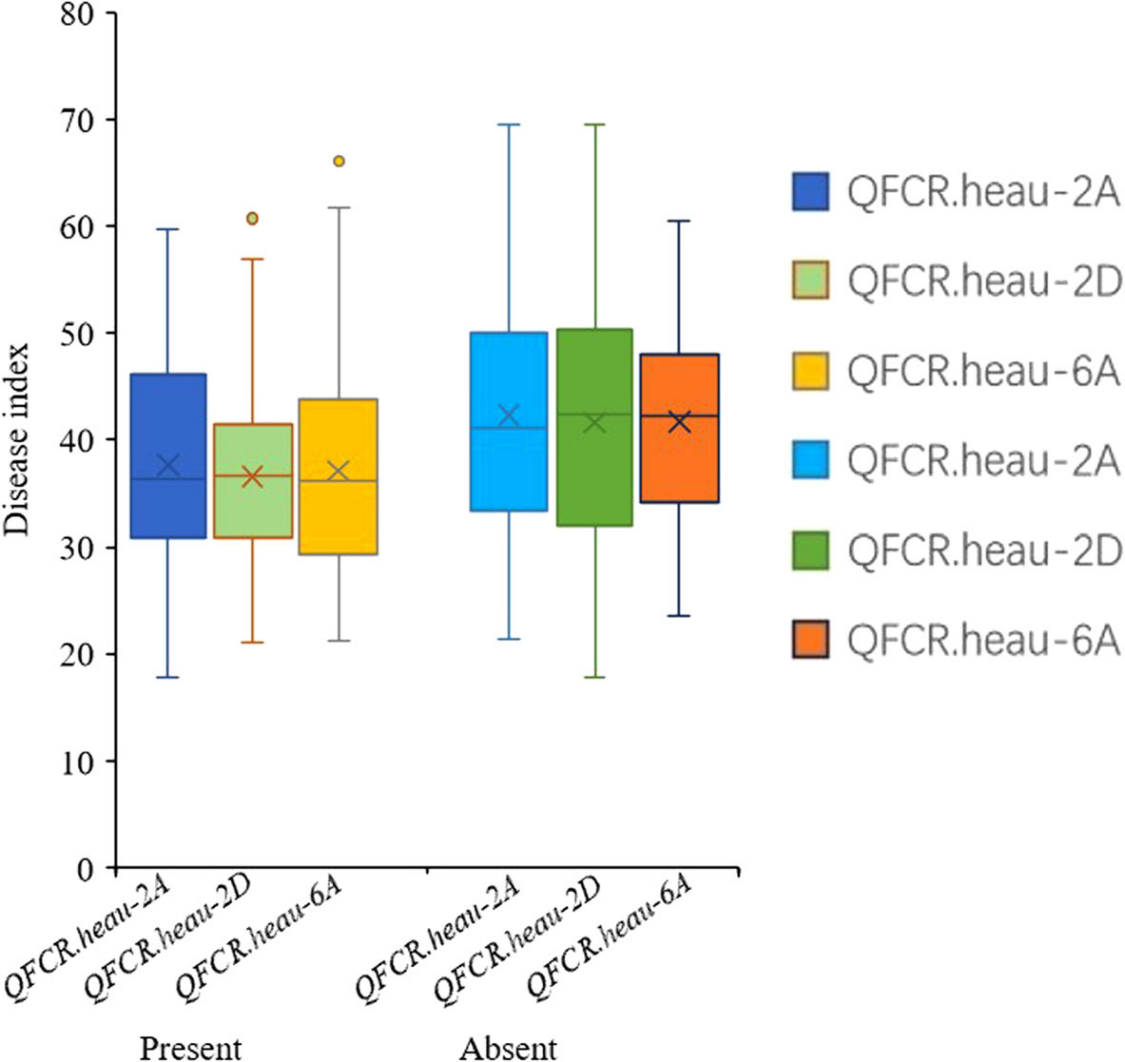
The averaged disease index of FCR resistance for the 3 QTLs present or absent

Three QTLs for FCR resistance were detected in the population derived from UC1110 (DI: 55.16%) and PI610750 (DI: 54.15%), which were significant in at least three of the four environments. The resistance QTLs on chromosomes 2D and 6A were derived from UC1110, whereas the one on chromosome 2A was from PI610750. The most stable QTL, designated as *QFCR.heau-6A*, was located between *wmc754-6A* and *barc1055* on 6A and was significant in all four environments (ZZ1, ZZ2, ZMD1, ZMD2), explaining 7.77–10.17% of the phenotypic variation (Table 4; Fig. 9a). Another QTL designated as *QFCR.heau-2D* and flanked by markers *cfd53* and *cfd43*, was significant in 3 environments (ZZ1, ZZ2, ZMD1) with phenotypic variation explained of 7.15–9.29% (Table 4, Fig. 9b). The QTL named as *QFCR.heau-2A* was between *gwm301* and *wPt-4861* and was significant in 3 environments (ZZ1, ZZ2, ZMD1), explaining 5.24–6.92% of phenotypic variation (Table 4; Fig. 9c). Further analysis of averaged DI of FCR for the 3 QTLs showed that wheat lines with *QFCR.heau-6A*, *QFCR.heau-2D* and *QFCR.heau-2A* displayed DI of 37.11, 36.51 and 37.67, respectively, whereas wheat lines without *QFCR.heau-6A*, *QFCR.heau-2D* and *QFCR.heau-2A* displayed DI of 41.71, 41.66 and 42.08, respectively (Fig. 10, Table 5). Furthermore, wheat lines with the three FCR-resistance QTLs showed the averaged disease index of 34.67, whereas those with none of the FCR-resistance QTLs showed an averaged DI of 47.44 (Table 5), and their differences reached extremely significant levels in each environment.

**Table 4:** QTL for FCR resistance in the UC1110 × PI610750 RIL population

QTL	Environment ^a^	Position (cM)	Left Marker	Right Marker	LOD ^b^	PVE (%) ^c^	Add ^d^
*QFCR.heau-2A*	ZZ1	8	*gwm301*	*wPt-4861*	2.92	6.92	3.10
	ZZ2	8	*gwm301*	*wPt-4861*	3.31	5.44	2.72
	ZMD1	8	*gwm301*	*wPt-4861*	3.15	5.24	2.68
*QFCR.heau-2D*	ZZ1	41	*cfd53*	*barc168*	3.99	9.29	−3.56
	ZZ2	43	*barc168*	*cfd43*	4.10	7.49	−3.17
	ZMD1	43	*barc168*	*cfd43*	3.91	7.15	−3.10
*QFCR.heau-6A*	ZZ1	82	*wmc754-6A*	*gpw2181-6A*	3.48	8.84	−2.21
	ZZ2	84	*barc3*	*barc1055*	4.31	10.17	−2.44
	ZMD1	82	*wmc754-6A*	*gpw2181-6A*	3.38	8.61	−2.18
	ZMD2	82	*wmc754-6A*	*gpw2181-6A*	3.07	7.77	−2.33

^a^ZZ1, 2015-2016Zhengzhou; ZZ2, 2016-2017Zhengzhou; ZMD1, 2015-2016Zhumadian; ZMD2, 2016-2017Zhumadian

^b^A threshold setting of 2.5 was used to declared the presence of a QTL

^c^Percent of phenotypic variance explained by the QTL

^d^Additive effects of the QTL, where positive values denote the origin of resistant alleles from PI610750 and negative values denote the origin of resistant alleles from UC111

**Table 5:** *t*-tests for the comparison of mean disease index of the resistance to FCR in UP population

QTL	Present/Absent ^a^	Number	ZZ1	ZZ2	ZMD1	ZMD2	Mean
*QFCR.heau-2A*	Present	46	37.72b	37.60b	37.69b	-^c^	37.67b
	Absent	101	42.58a	41.84a	41.81a	–	42.08a
*QFCR.heau-2D*	Present	55	37.35b	36.12b	36.09b	–	36.51b
	Absent	57	41.91a	41.53a	41.54a	–	41.66a
*QFCR.heau-6A*	Present	62	36.92b	37.14b	37.1b	37.28b	37.11b
	Absent	65	41.69a	41.54a	41.78a	41.83a	41.71a
*QFCR.heau-(2A + 2D + 6A)* ^*b*^	Present	5	35.29b	33.77b	34.22b	35.39b	34.67b
	Absent	20	47.79a	48.86a	48.99a	44.12a	47.44a

^a^The QTL present superior effect (present) or inferior effect (absent)

^b^The materials confer the three QTL at the same time

^c^The QTL not detected in the environment

## Discussion

FCR is a serious soil-borne disease in wheat and barley that not only causes yield loss but also brings health problem [4]. The pathogens of FCR (*Fusarium.* species) are tenacious fungus that can survive in stubble for more than 3 years which results in continued disease incidence [12]. Therefore, the genetic improvement of growing resistant and tolerant varieties is a key component in effectively managing the disease of FCR [36].

Previously, only limited efforts have been spent in screening sources of resistance and no cultivar showing fully resistance or immunity to FCR has been identified so far [2, 12]. Wildermuth and Puss [37] screened 400 wheat varieties for the resistance to FCR and found that only four showed moderate resistance. In the study of Zhang [38], there were only 13 varieties had moderate resistance to FCR, and most of the 82 tested varieties were susceptible. In this study, there was no immunity nor high resistance identified, and approximately 80% of the screened materials showed highly susceptible reactions to FCR. This was consistent with the result of Yang et al. [15] who performed a screening study on 88 wheat cultivars from the Yellow and Huai wheat region in 2015. Though the resistance resources to FCR are rare, it is helpful to divided the cultivars or advanced lines into different groups in terms of FCR resistance. In this regard, varieties such as Xunmai 118, Kaimai 26, Yanke 316, Xuke 732, Zhonglemai 9, Jinmai 1, Shenzhou 209, Fannong 1 and Jiyanmai 7 that showed stably moderate resistance (DI < 30) to FCR in multiple environments could be important resistant resources and should be considered for application in wheat FCR resistance breeding. Cultivars like Yanfeng 712, Yunong 805, Zhengmai 082, Jinmai 109 and Saidemai 7 that showed relative high susceptibility (DI >  70) to FCR in all environments should be avoid in FCR epidemic regions. In addition, cultivars like Fannong 3, Pumai 27, Nongda 399, Yunong 99 and Xuyou 46 that showed intermediate reactions (40 < DI < 60) should be paid sufficient attention to prevent FCR. Our study expanded the screened resources of FCR and highlighted a few resistant varieties for FCR resistance.

The accuracy of the phenotypic characterization for the disease over multi-environments are essential for assessing stability of FCR resistance and identification of stably expressed genomic regions [39]. So far, there is no uniform standard for the inoculation method and classification of resistance for wheat FCR [2, 4]. In this study, we used fungal colonized millet grains as inoculum to inoculate wheat seedlings, which has been considered to be the most reliable method to achieve an adequate level of FCR for obtaining consistent cultivar ranking [2, 40]. In addition, a key point in inoculation was to ensure that at least one millet grain was inoculated to a seedling, leading to the uniformity in infection and sufficient disease stress obtained in this study. This was reflected in the better correlation coefficient among experiments obtained in the current study (0.71 to 0.95) than in Erginbas-Orakci et al. [40] (*r*^*2*^ = 0.53) and Huo et al. [41] (*r*^2^ = 0.659). As seedling resistance is an important component of field performance [2], our seedling FCR experiments should be able to predicted the field results to a certain extent.

Up to date, FCR-resistant QTLs have been reported to distribute on 13 of the 21 chromosomes in wheat [2], but only QTLs on 2DL, 3BL and 5DS could be consistently detected in different genetic backgrounds [24]. Erginbas-Orakci et al. [20] identified that the QTLs related to crown rot resistance in CIMMYT spring wheat were located on chromosomes 3B and 2D with phenotypic effects of 11.4 and 11.6%, respectively. In this study, FCR-resistant QTLs were identified on 6A, 2D and 2A, and *QFCR.heau-6A* with a PVE of 7.77–10.17% between *wmc754-6A* and *barc1005* was a novel QTL because it was not reported previously. *QFCR.hau-2D* with PVE of 7.15–9.29% and *QFCR.hau-2A* with PVE of 5.24–6.92% could be detected in 3 environments, but they could be the same QTLs as reported in Zheng [24], Bovill et al. [42] and Martin [43]. These three QTLs showed significant additive effects (Fig. 10), and lines with all three QTLs exhibited good FCR resistance, implying the effectiveness of pyramiding all three QTLs to improve FCR resistance in wheat breeding programs.

In this study, significant SNPs closely associated with FCR were mainly distributed on chromosome 6A, e.g. AX-111106634, AX-94534539, AX-11170401, AX-109474774, AX-112290591, AX-111013769 and AX-110077933. The DI difference between cultivars with superior and inferior alleles at these SNP loci all reached extremely significant levels (*p* < 0.01). A total of 17 cultivars (i.e. Yanke 316, Jiyanmai 7, Jinmai 1, Zhonglemai 9, Shenzhou 209, Fannong 1, Hongmai 618, Zimai 627, Gengmai 237, Ximai 505, Zhoukang 918, Jinmai 108, Xuke 732, L 668, Xianmai 522, Yufeng 1 and Xinyanmai 98) with all superior alleles at these 7 SNP loci showed an average DI of less than 35, whereas 6 cultivars (i.e. Yunong 805, Zhengmai 082, Saidemai 7, Dongfanghongmai 6, Jinmai 109 and Wenmai 29) with all 7 inferior alleles at these loci showed an averaged DI of more than 70. Compared the DI of cultivars with only the 7 alleles (mentioned above) to the cultivars with 16 alleles from block1, the results of haplotypes *Hapl-1A* (a total of 16 superior alleles, including the 7 superior alleles) and *Hapl-1C* (a total of 16 inferior alleles, include the 7 inferior alleles) with the 7 alleles showed the same resistance to FCR with the results of 16 alleles. It suggested that these 7 alleles were with great effect to FCR and these loci usually closely linked and provided a guidance for pyramid breeding. It was consistent with the results of Sun [34] that revealed the superior alleles for pyramid breeding in 14 environments of 13 important agronomic traits in the Yellow and Huai valley of China.

Planting resistant cultivars is a valuable method to prevent FCR damage in wheat breeding program, and investigation of wheat FCR resistance requires effective and repeatable screening methods [44]. This study improved the inoculation method, screened some FCR-resistant wheat germplasm and identified some important FCR-resistant genetic loci that could be utilized in current elite cultivars. For example, we have identified several stable FCR-resistant genetic loci (i.e. AX-111106634, AX-94534539, AX-11170401, AX-109474774, AX-112290591, AX-111013769 and AX-110077933) and they could be utilized in marker-assisted selection in FCR disease resistance. Among these genetic loci, AX-111106634 on 6A was the most significant and stable locus.

QTL mapping indicated that the *QFCR.hau-6A* was located on 6A based on the genetic map of UC1110/PI610750. The physical position of the markers (≈ 495,246,396 bp) near the *QFCR.hau-6A* on 6A fell into the same chromosomal region as those detected from GWAS (490486046–497,462,135 bp) in the genome of Chinese Spring. Combination of GWAS and QTL mapping showed that the *QFCR.hau-6A* was the most significant QTL to modulate FCR resistance in this study and should be paid more attention to use for improvement of FCR resistance. All in all, both of the QTL mapping and GWAS in this study showed that there is an important genetic loci or QTL on the chromosome 6A with a relative largest effect of resistance to FCR, and thus this QTL could be considered as a priority for marker-assisted selection to improve FCR resistance in wheat cultivars from the Yellow and Huai wheat region. In addition, pyramiding several QTLs or genetic loci conferring partial FCR resistance in multiply environments by GWAS and QTL mapping will be able to improve FCR resistance for wheat pyramiding breeding.

## Conclusions

In this study, we investigated the FCR resistance of wheat cultivars or advanced lines from the Yellow and Huai wheat region and discovered some valuable FCR-resistant wheat germplasms. GWAS analysis showed that a total of 286 SNPs was significantly associated with FCR resistance in wheat, and 266 of them was on chromosome 6A. Physical mapping in a RIL population showed that there were 3 FCR-resistant QTLs, i.e. *QFCR.heau-6A, QFCR.heau-2D* and *QFCR.heau-2A.* The *QFCR.heau-6A* is a novel FCR-resistant QTL with the largest effect among them. Combination of GWAS and QTL mapping indicated that the FCR-resistant gene ranged from 490,486,046 to 497,462,135 (≈ 7.0 Mb) in Chinese Spring database.

## Methods

### Plant materials

A total of 234 common wheat cultivars or advanced lines were selected as an association panel for investigation of FCR resistance and GWAS analysis. They were released or developed after 2014, representing current breeding situation in the Yellow and Huai wheat region for the last 5 years. Seeds were harvested in Zhengzhou Scientific Research and Education Center of Henan Agricultural University (N 34.87°, E 113.60°) and Zhumadian Academy of Agricultural Science (N 33.01°, E 114.05°) during the cropping seasons 2015–2016 and 2016–2017.

A recombinant inbred lines (RIL) population with 187 F_10_ lines derived from UC1110 and PI610750 (UP) was used to map FCR resistance QTLs. Greenhouse experiments for this RIL population were the same as the GWAS panel.

### Phenotyping of Fusarium crown rot resistance

#### Fungal isolate

All plant materials were planted in greenhouse and inoculated with a prevalent Chinese *F. pseudograminearum* isolate namely WZ-8A, kindly provided by Prof. Honglian Li from the College of Plant Protection of Henan Agricultural University. WZ-8A is a highly aggressive strain isolated from infected crowns of wheat in northwest of the Yellow and Huai wheat region. The strain (culture medium with mycelium of 4 mm diameter) was put on a potato dextrose agar (PDA, 200 g of peeled potato, 15 g of agar and 20 g of dextrose in 1000 mL distilled water) plate at 25 °C under fluorescent lights with a 12 h/12 h day/night photoperiod in an incubator. After 6 days, when the white mycelium grew over the plates and showed peach-red to dark red pigmentation, a piece of 15 mm × 3 mm culture medium with mycelium was taken out from the margin of the plate, which was then transferred into a 2 mL centrifuge tube full of 30% glycerin and stored under − 80 °C for long-term storage. Another piece of medium with mycelium (of 4 mm diameter) was transferred into a 1.5 mL centrifuge tube with PDA medium and placed into the incubator to grow under the same condition mentioned above. After mycelia harvest, they were stored in 4 °C refrigerator for further application. When needed, mycelia from the 1.5 mL centrifuge tubes were transferred to fresh PDA plates for multiplication.

#### Spawn inoculum preparation

Millet grains of uniform size were boiled for 2 min and then rinsed 3–4 times with cold distilled water. The grains were put on clean gauze in fume hood until dry up and were then transferred into flasks for sterilization at 121 °C, 0.1 Mpa for 30 min. Shake the flasks before cooling down to prevent agglomeration. After cooling, a piece of medium with *F. pseudograminearum* mycelium was added to the flask, mixed thoroughly, and then placed in the incubator to grow at 25 °C under fluorescent lights with a 12/12 h day/night photoperiod. The flasks were shaken twice a day to promote uniform colonization. After 7 days, the colonized grains became ready for inoculation on seedlings.

#### Experimental design and inoculation

The experiments were carried out in plastic containers of 7 × 7 × 7 cm. Seeds were surface disinfested with 95% ethanol for 3 min and then rinsed under running distilled water for 3 min. Soil was sterilized at 121 °C for 1 h at 0.1 Mpa. When sowing, 150 g sterilized soil was put into a plastic container, followed by placing 12 sterilized seeds that were then covered with 20 g sterilized soil. Three replicates were performed for each cultivar. All plastic containers were placed on trays and watered from the bottom of the trays. The condition of the greenhouse was controlled at 25/20 °C day/night temperatures, 16 h/8 h light/dark photoperiod, and 60–80% relative humidity [45]. Three days after germination, weak seedlings were pulled out. When seedlings grew to 3 cm long, 8–10 seedlings per cultivar that grew uniformly were inoculated, then 0.4 g colonized millet grains (about 70–80 millet grains) were scattered to each plastic container. It is important to ensure at least one millet grain was distributed to the stem of a seedling. Then 30 g sterilized soil was scattered into each container and water was applied from the bottom of the trays. Subsequently, the containers were watered every two days.

#### Phenotyping

Severity of FCR was evaluated 4 weeks after inoculation, with a scale from 0 (no obvious symptoms) to 9 (completely necrotic damage) according to Li et al. [46] with some modification. Disease index (DI) was calculated for each replicate according to the methods of Li et al. [46] and Zheng et al. [24].

$$ \mathrm{DI}=\left(\sum {\mathrm{n}}_{\mathrm{s}}\ \mathrm{S}/9\ \mathrm{N}\right)\times 100 $$

Where, S is the scale value of each plant; n_s_ is the number of plants in the scale S; N is the total number of plants assessed in one replicate.

### Genotyping and quality control

All 234 accessions surveyed were genotyped using the wheat 660 K genotyping assay by Beijing CapitalBio Technology Company (http://cn.capitalbio.com/). The quality pretreatment of genotyping data was carried out for SNP call rate and MAF (minor allele frequency) with the PLINK software with threshold of --maf 0.02 and --geno 0.1. (http://zzz.bwh.harvard.edu/plink/tutorial.shtml) [47].

### Population structure analysis

Population structure was assessed by STRUCTURE software v2.3.4 with unlinked markers (*r*^2^ = 0) [48]. The number of subpopulations (k) was implemented by a burn-in of 1000 iterations followed by 1000 Monte Carlo Markov Chain (MCMC) replicates in a putative range of 1–10.

### Genome-wide association study

GWAS was implemented using the mixed linear model (PCA + K) by GAPIT packages in R software [49, 50] and the variance–covariance kinship matrix (K) was calculated using the VanRaden method [51]. Threshold for *P* value was calculated using a modified Bonferroni correction (Genetic type 1 Error Calculator, version 0.2) with a suggestive threshold of *P* value = 1.0e-4 (*P* = 1/n, n = effective SNP number) [52].

### Bi-parental QTL mapping

In total, 1494 polymorphic markers (SSRs, ESTs and DArTs) from 558 unique loci in the RIL population UC1110 × PI610750, kindly provided by Jorge Dubcovsky [53], were used to construct genetic linkage map by software IciMapping 4.0 (http://www.isbreeding.net). QTLs were calculated with the Inclusive composite interval mapping (ICIM) algorithm in four environments. The threshold of LOD scores was set at 2.5.

## Additional files

Additional file 1: **Table S1.** Resistance to FCR of wheat cultivars inoculated by *F.pg* in four environments. (XLSX 25 kb)

Additional file 2: **Table S2.** Information for FCR disease index and correlation coefficients among environments. (XLSX 9 kb)

Additional file 3: **Table S3.** ANOVA for FCR resistance of the GWAS panel. (XLSX 8 kb)

Additional file 4: **Table S4.** The most significant SNPs on chromosome 6A that highly correlation with FCR resistance in different environments. (XLSX 9 kb)

## Abbreviations

Add: Additive effects of the QTL
Chr.: Chromosome
cm: centimeter
DArT: Diversity arrays technology
DI: Disease index
DON: Deoxynivalenol
EST: Expressed sequence tags
FCR: Fusarium crown rot
GWAS: Genome-wide association studies
*hapl*: Haplotype
ICIM: Inclusive composite interval mapping
MAF: Minor allele frequency
MCMC: Monte Carlo Markov Chain
mm: millimeter
MSS: Most significant SNP
NIV: Nivalenol
PCA: Principal component analysis
PDA: Potato dextrose agar
PVE: Phenotypic variation explained
QTL: Quantitative trait loci
RIL: Recombinant inbred lines
SNP: Single nucleotide polymorphism
SSR: Simple sequence repeat
UP: UC1110 and PI610750
ZMD1: 2015–2016 Zhumadian
ZMD2: 2016–2017 Zhumadian
ZZ1: 2015–2016 Zhengzhou
ZZ2: 2016–2017 Zhengzhou
